# Supplementary material for: Frequency and perceived influence of menopausal symptoms on training and performance in female endurance athletes
Source: PLoS One. 2025 Dec 17;20(12):e0335738. doi: 10.1371/journal.pone.0335738 (PMC12711079; doi:10.1371/journal.pone.0335738)
Supplement: Attachment — Submitted filename: Review.docx [file pone.0335738.s002.docx]

# Reviewer’s Comment

The article addresses a highly relevant and globally significant topic, with recent scientific evidence highlighting physical activity as a key component in promoting health among menopausal women.

The manuscript focuses on the prevalence of menopause and the most commonly reported symptoms experienced during this phase, contributing to the understanding of the clinical and psychosocial impact of the menopausal transition. However, the introduction is unclear and lacks coherence. It fails to adequately contextualize the topic or provide foundational background, such as citing established guidelines from the North American Menopause Society (NAMS) and the International Federation of Gynecology and Obstetrics (FIGO). These guidelines emphasize the importance of regular aerobic and resistance physical activity in alleviating menopausal symptoms, improving quality of life, and reducing morbidity and mortality associated with common comorbidities.

Moreover, the introduction should include references that emphasize the relevance of the topic and identify existing gaps in the literature. For example, key conceptual definitions are only introduced later in the discussion section, which weakens the logical structure of the manuscript.

The study rationale must also be better justified. Research that investigates the impact of physical activity during menopause in greater depth is both urgent and highly relevant, especially considering the increasing life expectancy of women, growing concern with quality of life, and global population aging.

# Materials and Methods

Several methodological points need clarification:
- Exclusion criteria are not specified.
- Was surgical menopause considered in participant selection?
- Regarding the Menopause Rating Scale (MRS), it is recommended that the full 5-point Likert scale used in the study be described.
- The handling of missing data is unclear. It should be stated whether participants were required to complete all questions for the questionnaire to be considered valid.

# Results

This section requires several improvements for clearer and more effective presentation of the data:
- The statement “Prevalence of menopausal symptoms among female endurance athletes is high” must be clarified or rewritten to reflect the data accurately.
- Figures and tables present overlapping information. Therefore:
 - Figure 1 and Table 1 convey the same content; I recommend removing Figure 1.
 - Table 1 should present results stratified by menopausal status.
 - Figures 2, 3, 4, and 5 should be removed, as their data are already included in Table 2.
 - Table 2 requires improvement to enhance clarity and interpretability.

- In the paragraph stating “Several menopausal symptoms were reported to negatively affect training”, I recommend summarizing only the key findings, as the detailed information is already well presented in the accompanying table.

**Discussion**
The discussion is overly long and lacks comparative analysis with findings from other studies involving menopausal women.

While the authors provide a detailed account of the study's limitations, the manuscript would benefit from a more balanced perspective by also highlighting the strengths of the study. This would enhance the critical value and credibility of the discussion and contribute to the interpretation of the findings in a broader scientific context.
